# Expression of viral CD45 ligand E3/49K on porcine cells reduces human anti-pig immune responses

**DOI:** 10.1038/s41598-023-44316-y

**Published:** 2023-10-11

**Authors:** Claudia Pokoyski, Wiebke Baars, Mark Windheim, Thomas F. Reubold, Jasmin Zischke, Antje Brinkmann, Penelope C. Kay-Fedorov, Reinhard Schwinzer

**Affiliations:** 1 Surgical Research Laboratory, Hannover Medical School, Hannover, Germany; 2 Institute of Cell Biochemistry, Hannover Medical School, Hannover, Germany; 3 Institute for Biophysical Chemistry, Hannover Medical School, Hannover, Germany; 4 Institute of Virology, Hannover Medical School, and German Center for Infection Research (DZIF, TTU-IICH), Hannover-Braunschweig Site, Hannover, Germany

**Keywords:** Transplant immunology, Immune evasion

## Abstract

Transgenic expression of protective molecules in porcine cells and tissues is a promising approach to prevent xenograft rejection. Viruses have developed various strategies to escape the host’s immune system. We generated porcine B cells (B cell line L23) expressing the human adenovirus protein E3/49K or the human cytomegalovirus protein pUL11 and investigated how human T, NK and B cell responses are affected by the expression of the viral proteins. Binding studies revealed that E3/49K and pUL11 interact with CD45 on human but not porcine peripheral blood mononuclear cells. T cell proliferation in response to L23-E3/49K cells was significantly reduced and accompanied by development of an anti-inflammatory cytokine milieu (low: TNF-alpha, IFN-gamma, IL-6; high: IL-4, IL-10). Human peripheral blood mononuclear cells which had been primed for four weeks by L23-E3/49K cells included an extended population of regulatory T cells. Cytotoxicity of effector T and natural killer cells against L23 cells was significantly reduced (40 to 50%) by E3/49K expression. B cell activation and antibody production to E3/49K expressing cells was also diminished. Surprisingly, pUL11 expression showed no effects. Reduction of human anti-pig immune responses by transgenic expression of selected viral genes may be a novel approach for protection of porcine xenografts.

## Introduction

The availability of pigs carrying multiple genetic modifications was an important prerequisite for the recent achievements of long-term survival in preclinical pig-to-primate heart and kidney xenograft models^1,2^. Based on findings and knowledge gathered from these pig-to-primate models, the first life-supporting pig-to-human cardiac xenotransplantation and transplantation of pig kidneys in deceased human recipients have recently been performed^3–5^. The genome of source pigs used for these transplantations carried ten genetic modifications. Four resulted in the removal of porcine molecules (three immuno-dominant xenoantigen carbohydrates and the growth hormone receptor). Furthermore, six human transgenes (CD46, CD55, thrombomodulin, endothelial protein C receptor, heme oxygenase 1 and CD47) had been introduced to control the recipient’s complement and coagulation regulation system and prevent activation of endothelial cells and macrophages. Modifications specifically targeting the recipient’s T cell responses were not included in source pigs of the first clinical pig-to-human xenotransplants.

Basically there are two concepts whereby genetic modifications can be used to decrease human anti-pig T cell reactivity. First, porcine ligands can be deleted that trigger stimulatory signals when recognized by receptors on human T cells. Swine leukocyte antigen (SLA) molecules are promising candidates for the deletion strategy because they are recognized by human T cell receptors, which leads to strong activation of CD4^+^ and CD8^+^ T cells. Second, pig cells can be modified to express human ligands, which generate inhibitory signals by binding to receptors on human T cells. Examples for inhibitory ligands are the programmed cell death-1 ligand-1 (PD-L1, CD274) and human leukocyte antigen (HLA)-E. Binding of PD-L1 to the programmed cell death 1 receptor (PD-1, CD279) induces inhibitory signals which dampen T and B cell activation^6,7^. HLA-E is recognized by inhibitory receptors which impede the activation of human natural killer (NK) cells. SLA class I and class II deficient as well as PD-L1 and HLA-E transgenic pigs have been described, but information on functionality of these modifications in pig-to-primate transplantation models is still limited. Nevertheless, the efficiency of the concepts has clearly been confirmed by in vitro experiments^6–14^.

T cell responses to transplants can be divided into two different phases. During the induction or sensitization phase recipient T cells interact in the lymphatic tissue with source pig-derived antigen presenting cells that have migrated from the graft. Typical cellular consequences of human T cell receptor triggering by porcine SLA during the induction/sensitization phase are T cell proliferation and production of various cytokines. Proliferation and an appropriate cytokine milieu are required for the differentiation of CD8^+^ precursor cells to cytotoxic effector T cells (CTL). CTL leave the lymphatic tissue, migrate to the graft and destroy cells by different mechanisms. This scenario is known as the effector phase of an anti-graft response. We have recently shown that proliferation and cytokine production of human T cells stimulated with cells from SLA class I deficient pigs are reduced^9^. Cytotoxic activity of fully differentiated CD8^+^ CTL, however, was not influenced by SLA class I absence, suggesting that the requirements for effective control of the induction/ sensitization and effector phase are different. Following this idea, it is likely that inhibition of CTL cytotoxicity might be more difficult. This is also supported by data indicating the existence of FK506 resistant pathways of perforin-mediated cytotoxicity in CD8^+^ CTL^15^.

Although a great portion of cytotoxic activity derives from the CD8^+^ T cell subset, one has to keep in mind that CD4^+^ T cells likewise can develop cytotoxic potential after activation^16^. Cytotoxic CD4^+^ T cells have been described as important effector cells in immune responses to allo- and xenografts^17–19^. The perforin/granzyme pathway is regarded to be the primary cytotoxic mechanism of CD8^+^ effector cells. This pathway is also functioning in CD4^+^ effector cells. However, death receptor-dependent cytotoxicity seems to be more important. Thus, cytotoxic CD4^+^ T cells up-regulate ligands like FasL/CD95L of TNF-related apoptosis inducing ligand (TRAIL) which trigger apoptosis in target cells expressing corresponding receptors^20^. Because of the great variety of cytotoxic cells and mechanisms it will be important for clinical xenotransplantation to develop immunomodulatory strategies that inhibit cellular cytotoxicity during the effector phase of human immune responses to xenografts.

Viruses have developed immune evasion mechanisms to escape detection and elimination by the immune system of the host. Inhibition of the cytolytic capacity of effector cells is a robust viral strategy to prevent kill of virus-infected host cells. The mechanisms whereby two viral molecules, pUL11 and E3/49K, diminish immune responses of the host have recently been identified. Both the human cytomegalovirus (HCMV) glycoprotein pUL11 and human adenovirus type 64 (HAdV-D64 ; old taxonomy: Ad19a) protein E3/49K interact with the human cell surface protein tyrosine phosphatase CD45, which is essential for proper signaling via T and B cell antigen receptor associated pathways^21–23^. Binding of pUL11 and E3/49K or anti-CD45 antibodies is regarded to alter the phosphatase activity of CD45, thereby interfering with activating signaling pathways^24–30^. Inhibition of proliferation and cytokine production, enhanced production of immune attenuating cytokines (e.g. IL-10), but also suppression of cytotoxicity have been described as consequences of pUL11 or E3/49K-mediated alteration of CD45 function^21–23^. In this study we asked whether human anti-pig cellular immune responses can be reduced by recombinant expression of viral CD45 ligands. The data suggest that porcine cells expressing E3/49K do not only have a reduced capacity to trigger human T cell proliferation (induction phase), but show also reduced susceptibility to cytolysis by T and NK cells during the effector phase of a human anti-pig immune response.

## Results

### Genetically engineered pig cells expressing viral E3/49K and pUL11

**Figure 1:**
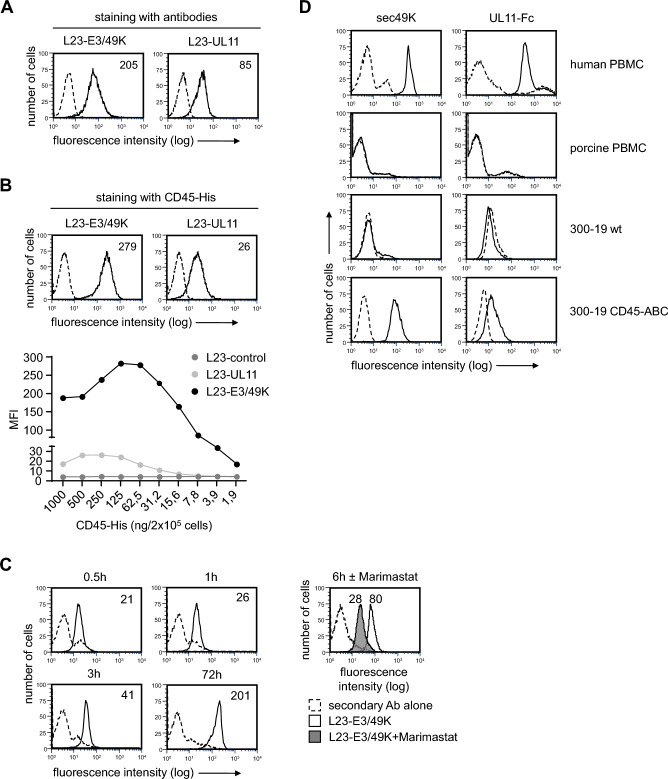
Characterization of viral E3/49K and pUL11 after transfection in porcine cells. (**A**) Expression in porcine L23 cells. L23-E3/49K (left) and L23-UL11 cells (right), shown as representative histograms, were stained with anti-E3/49K or anti-pUL11 monoclonal antibodies (mAbs), respectively (solid lines). Dashed-line histograms were obtained after staining of L23-control cells by the mAbs. The numbers represent mean fluorescence intensity. (**B**) Binding of CD45-His to transfected cells. Purified soluble CD45-His was used for staining of L23-E3/49K and L23-UL11 cells. Binding of soluble molecules to the cells was detected by incubation with PE-conjugated anti-CD45 mAb. Representative histograms show staining under optimal conditions. The numbers represent mean fluorescence intensity. The bar graph shows concentration dependent binding of CD45-His. The results of 1 of 2 similar experiments are shown. (**C**) Production of soluble E3/49K (sec49K) by transfected cells. Supernatants were harvested from L23-control cells (dotted line) or L23-E3/49K cells (solid line) at different time points after starting the culture. Human PBMC were incubated with supernatants followed by anti-E3/49K staining. Supernatants were also harvested from L23-E3/49K cells cultured for 6h without (solid line) or with Marimastat (filled histogram). The dashed line represents staining with secondary mAb alone. Representative histograms are shown and the numbers represent mean fluorescence intensity. (**D**) Binding of E3/49K and pUL11 to human CD45. Purified soluble E3/49K (sec49K-His) or pUL11 (UL11-Fc fusion protein) were used for staining of porcine and human PBMC, the mouse B cell line 300–19, and 300–19 cells transfected with the human CD45-ABC isoform. Binding of soluble molecules to the cells was detected by incubation with anti-E3/49K and anti-pUL11 mAb followed by a second incubation with fluorochrome-labelled secondary reagents. Dashed lines in the representative histograms represent background staining with the secondary antibodies alone.

The porcine B cell line L23 was transfected with vectors coding for the HAdV-D64 protein E3/49K or the HCMV protein pUL11. Stable cell lines were established and expression of the viral proteins was assessed by flow cytometry. Staining with antibodies to E3/49K or pUL11 revealed cell surface expression of the two molecules in transfected cells but not in L23-mock controls (Fig. 1A). However, fluorescence intensities obtained with anti-E3/49K antibodies were always enhanced implying higher expression levels of E3/49K compared to pUL11. Additional experiments using soluble CD45 (CD45-His) were performed to directly compare the relative expression levels of the two proteins. These experiments confirmed greater amounts of E3/49K (Fig. 1B). A soluble form of E3/49K, sec49K, has been described in human cells^23^, which is generated by matrix metalloprotease-mediated cleavage of membrane-bound molecules^31^. We studied whether this occurs also in transfected pig cells. L23-E3/49K and L23-control cells were washed and seeded in fresh medium. Supernatants from growing cultures were collected at different time points and incubated with human peripheral mononuclear cells (PBMC). Binding of soluble E3/49K to human PBMC could be detected already after 0.5 h by using anti-E3/49K mAb (Fig. 1C). The level of soluble E3/49K continuously increased until the final analysis after 72 h. No staining of human PBMC was observed when supernatants from L23-control cells were used (Fig. 1C). When L23-E3/49K cells were cultured in the presence of the metalloprotease inhibitor Marimastat, the level of soluble E3/49K in the supernatant was reduced (Fig. 1C), suggesting that cleavage of membrane bound molecules is mediated by metalloproteases like in human cells^31^.

Genetic modifications, which are carried out to optimize a xenograft for clinical usage, should have no deleterious effects on survival and maintenance of source pigs. Both human HAdV-D64 and human HCMV are regarded to act species-specifically^21,23^. To study whether this applies also for the recombinant proteins E3/49K and pUL11, we tested binding on porcine and human PBMC. The purified soluble sec49K-His and a recombinant soluble pUL11-Fc were used for these experiments. Human PBMC were strongly labelled by both, but neither one reacted with porcine PBMC (Fig. 1D). Further studies using mouse cells (300–19) transfected with human CD45 confirmed earlier data^21,23^ showing that human CD45 is the binding partner of E3/49K and pUL11 (Fig. 1D).

### Human PBMC respond with reduced proliferation to pig cells expressing viral E3/49K

**Figure 2:**
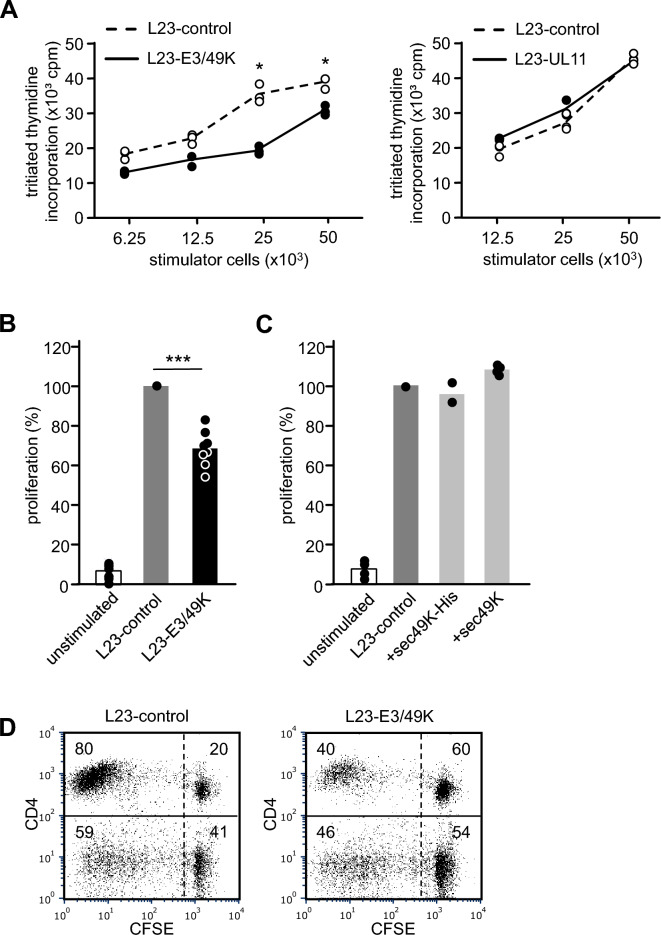
Influence of viral E3/49K and pUL11 on human PBMC proliferation. (**A**) Assessment of PBMC proliferation by tritiated thymidine incorporation. Human PBMC (1 × 10^5^/well) were stimulated with increasing numbers of irradiated L23-control, L23-E3/49K or L23-UL11 cells. The cells were cultured for 5 days, pulsed with tritiated thymidine and harvested after 16 h. One representative experiment out of a series of eight and four is shown comparing the stimulatory capacity of L23-E3/49K and L23-UL11 cells, respectively, with L23-control cells. Results are expressed as mean cpm of triplicate cultures. Statistical analysis was performed by applying the paired Student’s *t*-test. (**B**) Proliferative responses of PBMC from eight different human blood donors to stimulation with L23-control and L23-E3/49K cells. 1 × 10^5^ human PBMC were stimulated with 25 × 10^3^ irradiated L23 cells. Proliferation was determined after 6 days. Results are expressed as percent proliferation relative to L23-control cells (100%). Statistical significance was determined by ANOVA. (**C**) Studies using soluble E3/49K. Human PBMC (1 × 10^5^/well) were stimulated with irradiated L23-control cells (25 × 10^3^/well). Culture was performed without soluble E3/49K (unstimulated) or in the presence of purified soluble E3/49K (sec49K-His; n = 2) or supernatant from L23-E3/49K cells (sec49K; n = 4). The cells were cultured for 5 days, pulsed with tritiated thymidine and harvested after 16 h. Results are expressed as percent proliferation relative to cultures without E3/49K (100%). (**D**) Assessment of T cell proliferation by CFSE dilution. Human PBMC (1 × 10^5^/well) were stained with CFSE and stimulated with irradiated L23-control or L23-E3/49K cells (25 × 10^3^/well). The cells were cultured for 7 days, stained with anti-CD4-mAb and analyzed by flow cytometry. The numbers represent % positive cells in each quadrant of the representative histograms.

The effect of E3/49K and pUL11 expression on the xeno-stimulatory capacity of porcine cells was studied by mixed lymphocyte reaction (MLR) experiments. L23-control cells triggered strong proliferation of human PBMC (Fig. 2A). Proliferation was diminished when L23-E3/49K cells were used for stimulation. Expression of pUL11, however, had no effect on the stimulatory potential of L23 cells. In a series of experiments studying human PBMC from 8 different blood donors, proliferation triggered by L23-E3/49K cells was reduced by about 30% compared to L23-control cells (Fig. 2B). To study a possible role of soluble E3/49K in this system, we stimulated human PBMC with L23-control cells and added purified sec49K (sec49K-His) or supernatant from L23-E3/49K (sec49K) to the cultures. Proliferation was not altered by soluble molecules (Fig. 2C). Decreased proliferation in response to L23-E3/49K cells was observed in the CD4 and the CD8 T cell subset. 80% of CD4^+^ T cells proliferated to stimulation with L23-control cells but only 40% to L23-E3/49K cells (Fig. 2D). Among CD8^+^ (CD4^-^) T cells proliferation was reduced from 59 to 46% (Fig. 2D).

### Human PBMC responding to L23-E3/49K cells show altered p56lck(p505) phosphorylation

**Figure 3:**
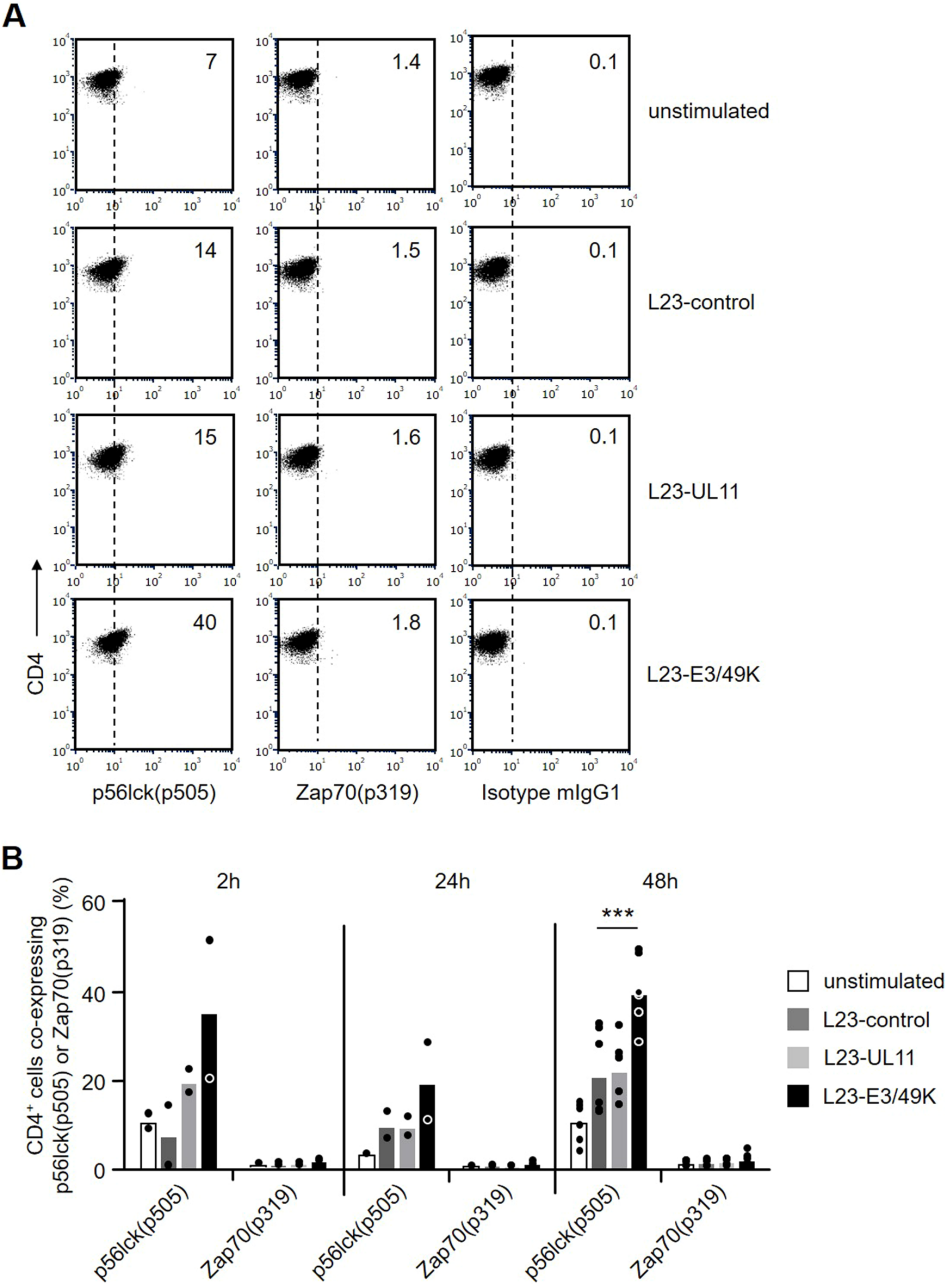
Phosphorylation of p56lck(p505) and Zap70(p319) in PBMC stimulated with L23-transfectants. (**A**) PBMC that had been stimulated for 48 h with L23-control, L23-UL11 or L23-E3/49K cells were intracellularly stained with p56lck(p505) or Zap70(p319) in combination with anti-CD4 and analyzed by flow cytometry. Dot plots show reactivity of p56lck(p505) and ZAP70(p319) in gated CD4^+^ cells and were obtained in a representative experiment. The numbers represent percent positive cells. (**B**) The bar graph summarizes mean percentages of CD4^+^p56lck(p505)^+^ and CD4^+^Zap70(p319)^+^ cells from 2 (2 h), 2 (24 h) or 6 (48 h) independent experiments. Statistical significance for the p56lck(p505) data at 48 h was determined by ANOVA.

An appropriate level of CD45 phosphatase activity is required to ensure proper signaling via T and B cell antigen receptors. Thus, we asked whether reduced proliferation of human PBMC in response to L23-E3/49K cells may result from impaired CD45 phosphatase activity due to E3/49K binding. A well known target of CD45 is the tyrosine kinase p56lck which is an essential component of T cell receptor signaling pathways. The enzyme is inactive when tyrosine-phosphorylated at residue 505 and can be activated by CD45-mediated dephosphorylation of this residue^32^. Thus, we assumed that strong CD45 phosphatase should be accompanied with low level p56lck(p505) phosphorylation whereas diminished phosphatase activity should result in enhanced p56lck(p505) phosphorylation. After 48 h stimulation of PBMC with L23-control or L23-UL11 cells, 15–20% of CD4^+^ T cells possessed phosphorylated p56lck(p505) (Fig. 3A). When L23-E3/49K cells were used for stimulation, phosphorylated p56lck(p505) was detected in approximately 40% of CD4^+^ T cells. Enhancement of p56lck(p505) phosphorylation by E3/49K was also observed at earlier time points (2 h and 24 h; Fig. 3B). This enhanced phosphorylation supports the idea that binding of E3/49K to CD45 reduces its phosphatase activity thereby generating a greater pool of inactive p56lck. This is in line with recently published data using purified sec49K for modulation of CD45 activity^33^. We also studied Zap70, another molecule involved in T cell receptor signaling. Neither early after stimulation nor after 48 h any alterations in phosphorylation of Zap70 residue 319 could be detected (Fig. 3B).

### Human PBMC responding to L23-E3/49K cells generate an anti-inflammatory cytokine milieu

**Figure 4:**
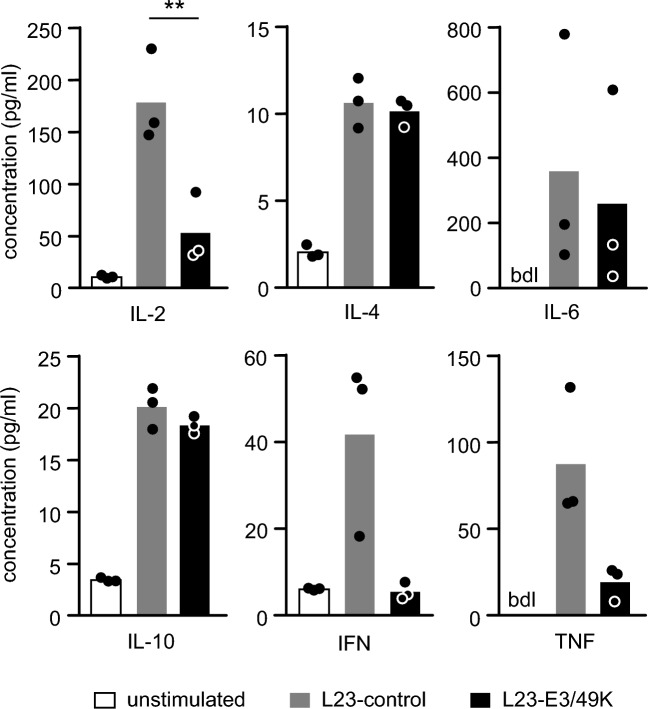
Effect of viral E3/49K on cytokine levels of stimulated human PBMC. Human PBMC (1 × 10^5^/well) were stimulated with irradiated L23-control or L23-E3/49K cells (25 × 10^3^/well). Supernatants of the co-cultures were harvested after 24h and analyzed for levels of IL-2, IL-4, IL-6, IL-10, IFN-γ and TNF-α. The bar graph summarizes means of 3 experiments (concentration; pg/ml). Statistical significance was determined by ANOVA.

To further characterize human PBMC reactivity to L23-E3/49K cells, we studied the cytokine patterns in these cultures. Compared to stimulation with L23-control cells, the levels of growth-promoting IL-2 was significantly diminished and the pro-inflammatory cytokines IL-6, interferon (IFN)-γ, and tumor necrosis factor (TNF)-α were considerably reduced (Fig. 4). In a series of experiments using responder PBMC from 3 different human blood donors IL-2, IFN-γ and TNF-α were reduced by 70 to 80% and IL-6 by approximately 30%. On the other hand, production of the pleiotropic cytokine IL-4 and the anti-inflammatory cytokine IL-10 was not affected by expression of E3/49K on L23 cells. Together, this data suggests that the cytokine pattern of human anti-L23-E3/49K PBMC responses corresponds to an anti-inflammatory cytokine milieu.

### E3/49K-treated human PBMC develop an unusual activation state in the long-term course

**Figure 5:**
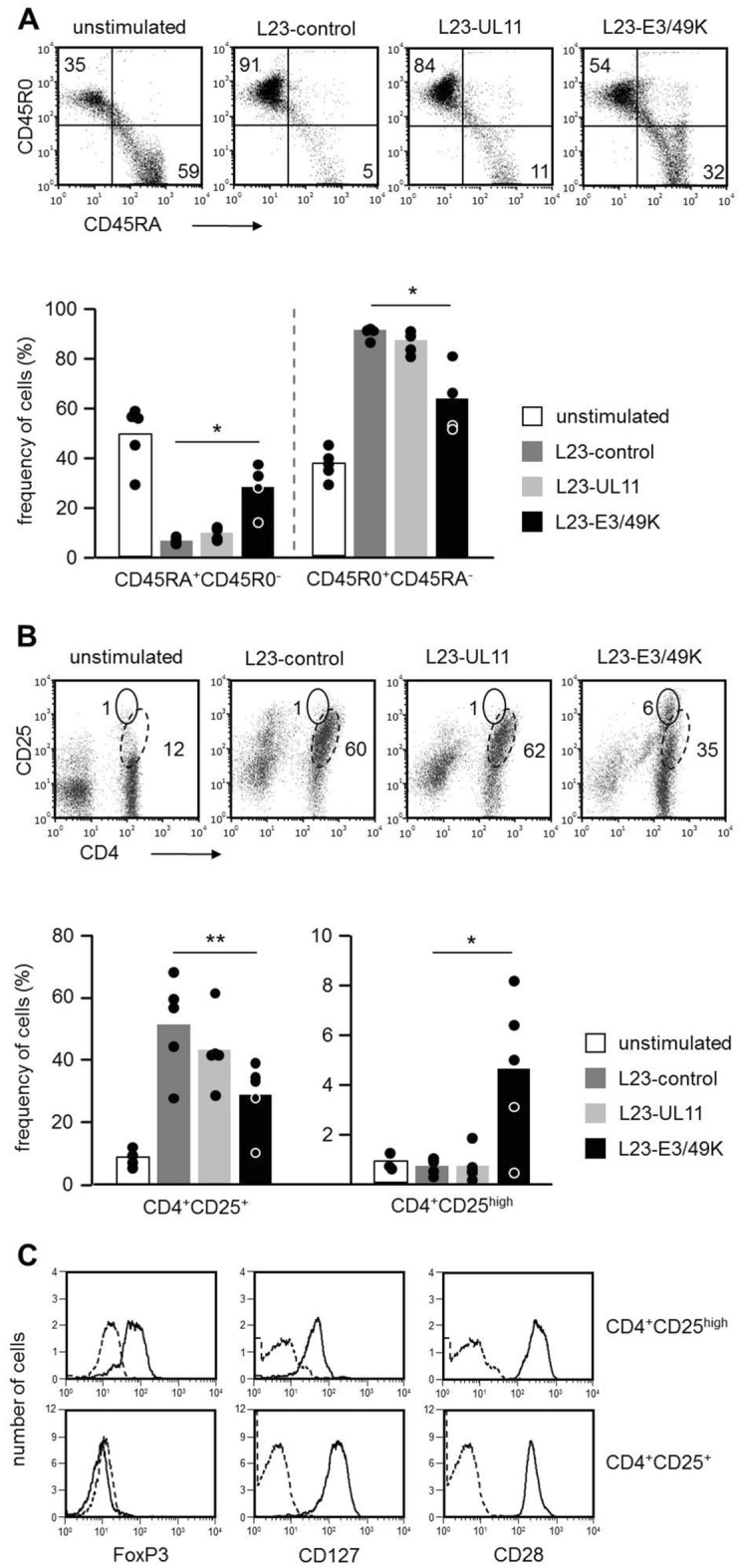
Phenotypic characterization of human PBMC after chronic stimulation with L23 cells. Human PBMC were analyzed by flow cytometry on day 0 (unstimulated) and on day 28 after repetitive re-stimulation (every week plus fresh medium) using irradiated L23-control, L23-UL11, and L23-E3/49K as stimulator cells. (**A**) Activation induced alterations of CD45RA and CD45R0 isoforms. The cells were stained by CD45RA in combination with CD45R0 mAb. Forward-/side-scatter gating was performed to exclude porcine L23 cells from the analysis. Data presented as dot plots of CD45RA/CD45R0 co-expression were obtained in a representative experiment. The numbers represent the percentage of CD45RA^+^CD45R0^-^ (lower right quadrant) and CD45R0^+^CD45RA^-^ (upper left quadrant) cells. The bar graph summarizes percentages from 5 independent experiments. Statistical significance was determined by ANOVA. (**B**) Activation induced upregulation of CD25. The cells were stained by CD4 in combination with CD25 mAb. Forward-/side-scatter gating was performed to exclude porcine L23 cells from the analysis. The dot plots show CD4/CD25 co-expression patterns obtained in a representative experiment. CD4^+^ T cells expressing intermediate levels of CD25 are indicated by dashed ellipses, CD4^+^CD25^high^ cells by solid ellipses. The numbers represent the percentage of cells in the ellipses. The bar graph summarizes percentages from 5 independent experiments. Statistical significance was determined by ANOVA. (**C**) Characterization of CD4^+^CD25^high^ cells. Chronically L23-E3/49K-stimulated cells were stained by CD4 and CD25 in combination with either FoxP3, CD127 or CD28 mAbs. Dashed lines represent isotype controls (FoxP3) or secondary antibodies alone. The histograms were obtained in a single experiment and were confirmed in a second experiment using cells from another blood donor.

To study the long-term consequences of an exposure to viral ligands, we cultured human PBMC with L23-transfectants for four weeks by weekly re-stimulation and analysed the resulting T cell population by flow cytometry. To provide optimal conditions, culture medium was replaced each week when PBMC were re-stimulated. Furthermore, we monitored cell growth by weekly cell counting. Within one week (day 0 to 7, day 7 to 14, day 14 to 21, or day 21 to 28) cell numbers of PBMC stimulated with L23-control or L23-UL11 cells increased by approximately 30%. In week one and two, a small but reproducible cell growth was also found in PBMC stimulated with L23-E3/49K cells (e.g. 3–4% increase from day 7 to day 14). In contrast, there was a cell loss at later time points (approx. 35% decrease from day 21 to 28). Since both pUL11 and E3/49K bind to CD45 (Fig. 1C), we first asked whether the activation induced switch from CD45RA to CD45R0 isoforms^34,35^ is altered by binding of pUL11 or E3/49K. In unstimulated human PBMC, about 50–60% of cells expressed CD45RA but no CD45R0 (CD45RA^+^CD45R0^-^ cells) and about 30–40% were CD45R0^+^CD45RA^-^ (Fig. 5A). The great majority (80 – 90%) of human PBMC co-cultured with L23-control or L23-UL11 cells had lost CD45RA isoforms and instead expressed CD45R0. In contrast, in human PBMC/L23-E3/49K co-cultures we found a high frequency of CD45RA^+^CD45R0^-^ cells and lower proportion of CD45R0^+^CD45RA^-^ suggesting that activation induced CD45 isoform switch is less pronounced. Thus, we concluded that T cells stimulated in the presence of E3/49K do not undergo complete activation/differentiation.

To characterize further the activation/differentiation stage of human PBMC after chronic in vitro stimulation, we monitored CD25 expression levels on CD4^+^ T cells. Compared to unstimulated human PBMC, CD25 was upregulated to intermediate levels after co-culture with L23 cells (Fig. 5B). In human PBMC stimulated with L23-control and L23-UL11 cells, 45–65% of cells expressed a CD4^+^CD25^+^ phenotype (Fig. 5B, dashed ellipses). After L23-E3/49K stimulation, however, this population was significantly reduced, indicating a diminished level of activation. Interestingly, in these cultures a distinct CD4^+^ cell population appeared expressing the phenotype of T_reg_ (CD4^+^CD25^high^) (Fig. 5B, solid ellipses). CD4^+^CD25^high^ cells were almost absent in unstimulated human PBMC and human PBMC cultured for 4 weeks with L23-control or L23-UL11 cells. CD4^+^CD25^high^ cells co-expressed FoxP3 and low levels of CD127 supporting their classification as T_reg_ (Fig. 5C). As mentioned above, the yield of viable cells in human PBMC/L23-E3/49K cultures was particularly low impeding further studies characterizing the functional spectrum of human PBMC after long-term E3/49K stimulation. Preliminary data indicated that this population is hypo-reactive when re-stimulated with L23-control cells in a secondary response (data not shown).

### L23-E3/49K cells are partially protected from cytotoxicity by human effector cells

**Figure 6:**
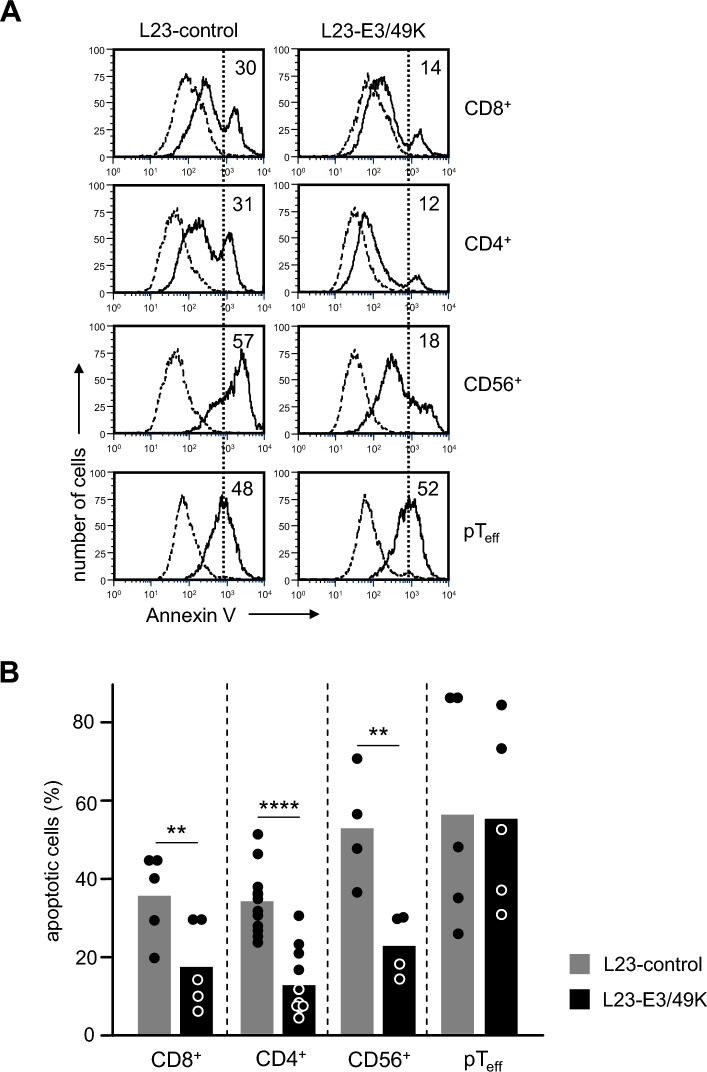
Susceptibility of porcine target cells to cell death induced by human cytotoxic effector cells. A total of 1 × 10^5^ porcine target cells (L23-control and L23-E3/49K) were co-cultured with 1 × 10^5^ human cytotoxic effector cells. After 2 h the cells were washed and stained with a combination of monoclonal antibody W6/32 (anti-human HLA class-I) and Annexin V. Analysis of Annexin V staining was performed in “gated” porcine L23 cells as defined by absence of W6/32 staining. (**A**) Representative histograms of Annexin V staining in L23-control and L23-E3/49K cells alone (dashed lines) or after co-cultures with human anti-L23 sensitized T cell lines (CD8^+^ or CD4^+^), CD56^+^ NK and NKT cells or porcine CTL generated in vitro by allogeneic mixed lymphocyte reaction (pTeff) (solid lines). The dotted line discriminates viable (Annexin V^low^) and apoptotic (Annexin V^high^) cells. Numbers represent the proportion of apoptotic/dead L23 cells. (**B**) The bar graph summarizes percentages of apoptotic cells from 5 different experiments using cells from 2 blood donors (CD8^+^), 12 experiments with cells from 2 donors (CD4^+^), 4 experiments using cells from 3 different blood donors (CD56^+^), and 5 experiments with cells from 2 donors (pTeff). Statistical analysis was performed by applying the paired Student’s *t*-test.

Next, we addressed the question as to whether expression of viral proteins on porcine cells influences their susceptibility to cellular cytotoxicity. Human CD8^+^ and CD4^+^ T cells sensitized in vitro to SLA molecules of L23 cells were used as cytotoxic effectors, as well as IL-2 activated CD56^+^ NK and natural killer T (NKT) cells. Furthermore, porcine allogeneic effector T cells (pT_eff_) were generated by in vitro stimulation of porcine PBMC with L23 cells. Annexin V binding assays were applied to detect apoptotic cell death among the L23 target population. A representative experiment is depicted in Fig. 6A. CD8^+^ T cells induced apoptosis (Annexin V high phenotype) in 30% of L23-control cells whereas only 14% of L23-E3/49K cells were killed. Similarly, the capacity of CD4^+^ T cells and CD56^+^ NK and NKT cells to induce apoptosis in porcine target cells was significantly reduced by E3/49K expression. The summary of these experiments shows that E3/49K expression reduced cell death of L23 cells by about 40 to 50% (Fig. 6B). On the other hand, porcine effector T cells (pT_eff_) killed L23-control and L23-E3/49K cells with similar intensity. This observation supports the assumption that HAdV-D64 is species(human)-specific and is in line with binding of E3/49K to human but not porcine cells (Fig. 1C). We also analyzed the protective capacity of pUL11. However, expression of pUL11 did not significantly decrease susceptibility of L23 cells against cytotoxic effectors (data not shown).

### L23-E3/49K-stimulated B cells show diminished activation and antibody production

**Figure 7:**
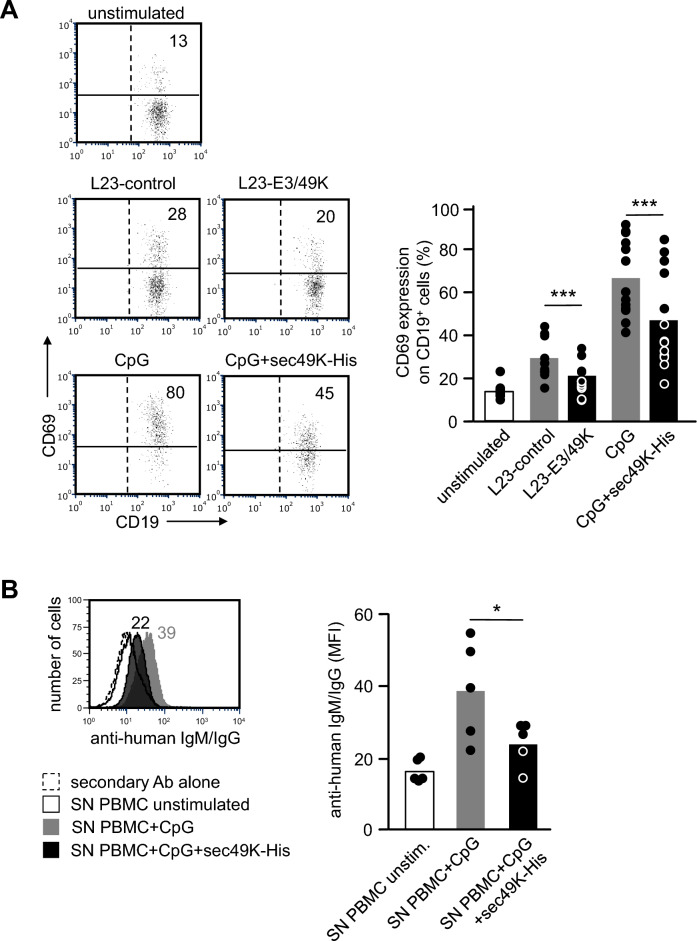
Effect of E3/49K on B cell activation and antibody production. (**A**) Assessment of B cell activation by CD69 expression. Human PBMC (2 × 10^5^/well) were cultured without stimulus, with L23-control or L23-E3/49K cells, with CpG alone, or with CpG plus purified soluble E3/49K (sec49K-His). After 24 h, the cells were stained with the antibody combination CD19/CD69 and analyzed by flow cytometry. A representative experiment showing dot-plots of CD19/CD69 co-expression patterns is depicted. Numbers are percentages of CD69^+^ cells among the CD19^+^ B cell population. The bar graph summarizes percentages of CD19^+^CD69^+^ cells obtained in 13 independent experiments. Statistical significance was calculated by ANOVA. (**B**) Demonstration of in vitro antibody production. Human PBMC were cultured for 11 days with medium, or stimulated with CpG alone or CpG in the presence of soluble E3/49K. Supernatants (SN) were harvested and titrated on porcine L23-control cells. The presence of antibodies binding to L23 cells was visualized by a second incubation step using FITC-labelled goat anti-human IgM and IgG antibodies and flow cytometry. The numbers in the representative histogram represent mean fluorescence intensity. The bar graph summarizes means obtained in a series of experiments using cells from 5 different blood donors. Statistical significance was calculated by ANOVA.

Because CD45 is strongly expressed also on B cells, we asked whether B cell activation might be influenced by E3/49K. When human PBMC were cultured for 24 h with L23-control or L23-UL11 cells or the polyclonal B cell activator CpG, strong B cell activation was observed as demonstrated by significant upregulation of CD69 on CD19^+^ B cells (Fig. 7A). In the presence of L23-E3/49K cells or soluble E3/49K, however, the proportion of CD19^+^ B cells expressing CD69 was markedly reduced. In a series of experiments using PBMC from 13 different human blood donors, B cell activation—as monitored by CD69 expression—was diminished by about 25 – 30% (Fig. 7A, bar graph).

Humans have a high frequency of memory B cells producing antibodies to alpha-Gal epitopes^36^. Thus, we assumed that polyclonal in vitro B cell activation by CpG might induce detectable levels of anti-pig antibodies. Indeed, in supernatants from human PBMC cultured for 11 days with CpG, antibodies to L23 cells were readily detected (Fig. 7B). The level of such antibodies was significantly reduced when CpG stimulation was performed in the presence of soluble E3/49K (Fig. 7B, bar graph). Together these observations suggest that targeting of human CD45 by recombinant viral ligands could be a promising strategy not only to reduce human T cell responses to xenograft but also to diminish anti-pig humoral immunity.

## Discussion

CD45 has been discussed to be an interesting target for immune intervention^37^. The identification of the viral proteins E3/49K and pUL11 as specific ligands for CD45 offered the opportunity to use these molecules for immunomodulation. Here we show that recombinant expression of E3/49K and pUL11 is feasible in porcine cells (Fig. 1A). Studying immune responses in vitro revealed that human PBMC responded to E3/49K expressing porcine transfectants with reduced proliferation suggesting diminished T cell responses and altered TcR signaling. Furthermore, generation of an anti-inflammatory cytokine milieu, expansion of CD4^+^CD25^high^FoxP3^+^ T_reg_, and reduced cytotoxicity were observed. In addition, the upregulation of CD69 on B cells and the antibody production in response to stimulation with E3/49K expressing cells was diminished. These findings will be discussed in the context of the current knowledge of viral immune escape mechanisms. Furthermore, the question as to whether the “viral approach” can be applied to protect porcine grafts in clinical xenotransplantation will be addressed.

Human adeno- and cytomegaloviruses (HAdV, HCMV) are able to persist in the human body and subvert antiviral immune responses, because their genomes contain genes that encode proteins with immunomodulatory functions^21,38,39^. Chronic viral infections can lead to decay in the number of T cells, functional exhaustion of T cells, and impaired cytokine production (loss of IL-2, TNF-α and IFN-γ)^40,41^. In particular for the viral CD45 ligand E3/49K, it has been shown that the molecule suppresses T cell activation and signaling, decreases production of the antiviral cytokine IFN-γ, and inhibits cytotoxicity of NK cells^23^. Furthermore, it has been suggested that the immunosuppressive environment created by latent viruses may induce the activation of T_reg_ which downregulate the host’s anti-viral immune responses thereby maintaining viral persistence^42^. Our in vitro data confirm that typical T cell functions like proliferation and signaling (Figs. 2 and 3), cytokine synthesis (Fig. 4), and cellular cytotoxicity (Fig. 6) can be diminished by expression of E3/49K on porcine L23 cells. This is in line with the assumption that the T cell compartment is the major target of viral escape mechanisms. The observation that B cell activation and antibody production to L23 cells was significantly reduced by recombinant expression of E3/49K (Fig. 7) strongly suggests that binding of E3/49K to CD45 on B cells is associated with signaling events that diminish B cell responses. Thus, it is likely that viral escape mechanisms not only aim at decreasing the host’s T and NK cell responses, but in addition act on B cells to reduce anti-viral antibody responses.

It has been shown, that the interaction between pUL11 and CD45 inhibits the phosphatase activity of CD45 and results in impaired T cell receptor signaling and T cell proliferation^22,43^ as well as in an increase in production of the anti-inflammatory cytokine IL-10^22^. Surprisingly, expression of pUL11 on porcine L23 cells had very little, if any, effects on human anti-L23 PBMC proliferation (Fig. 2A). E3/49K, on the other hand, effectively decreased human T cell responses. Chronic viral infections with HCMV are essentially silent with a total lack of new virus production^44^. Therefore, pUL11 may have a different function during viral latency than E3/49K and that could be one reason why we did not find an effect of pUL11 in our experiments. Another reason for the lack of functionality of pUL11 in our experimental setting could be low level expression of pUL11 on L23 cells (Fig. 1B). The cell surface density of CD45 molecules seems to be a critical factor for its functional activity and can influence immune responses positively or negatively^22,45^. Depending on its concentration, pUL11 has been shown to mediate activating or inhibitory effects on CD45 phosphatase activity^22^. Since inhibitory effects require high concentrations the expression level of pUL11 on L23-UL11 cells may be too low to trigger inhibitory effects. E3/49K and pUL11 presumably do not have the same binding site on the CD45 molecule, because neither can block the other from binding to CD45 (data not shown). Thus, binding of E3/49K and pUL11 to CD45 may differentially modify its phosphatase activity and thereby the intensity of functional alterations in target cells.

L23-E3/49K cells expressed high levels of E3/49K on the cell surface and the molecule was also proteolytically cleaved, leading to the secretion of the ectodomain sec49K (Fig. 1A, C). Our data suggested that inhibition of T cell functions (e.g. proliferation, signaling) mainly results from the activity of membrane bound E3/49K whereas purified soluble sec49K as well as the supernatant of L23-E3/49K cells had no inhibitory effects on T cells (Fig. 2C). However, B cell activation and antibody production were significantly inhibited by purified soluble sec49K and also by membrane bound E3/49K (Fig. 7). Detailed knowledge of the modulatory functions of membrane bound E3/49K and soluble sec49K is required for possible usage of these molecules as protective transgenes in clinical xenotransplantation. Cleavage of sec49K takes place on the cell surface and is conducted by matrix metalloproteases (Fig. 1C). Experiments are underway to generate a non-cleavable mutant of the E3/49K protein which can be expressed in porcine L23 cells. Comparing immune functions against our current L23-E3/49K cells (membrane and soluble E3/49K) and L23 transfectants exclusively expressing membrane bound E3/49K will help to distinguish between the functional spectrum of the two types of E3/49K.

If proteins derived from HAdV of HCMV should be used as transgenes to protect porcine xenografts from human immune responses one has to be aware of the fact that many individuals are immunized against HAdV and HCMV. Thus, it has to be assumed that anti-viral antibodies and memory T cells may be present in some recipients of xenografts. The frequency of individuals with neutralizing antibodies to HCMV is 66% in the European region^46^ and a seroprevalence of 16–19% was reported toward HAdV-D64^47,48^. It is difficult to assess whether anti-E3/49K or anti-pUL11 antibodies are generated during immune responses induced by HAdV or HCMV. However, there are some published data arguing against this possibility. Both, E3/49K and pUL11 are viral proteins that mask infected cells from recognition by the host’s immune system^49,50^. The human anti-HCMV immune reaction, however, is mainly directed against proteins of the viral capsid (pp65, gB,gH) and not against masking proteins^49^. Furthermore, it has been shown that the pUL11 protein appears on the cell surface 48 h after infection, but is expressed in very low density and is not capable of mediating ADCC in vitro^51^. There is not much data available regarding the neutralizing epitopes of the more than 50 human HAdV serotypes. Nevertheless, major targets of adenovirus neutralizing antibodies seem to be three major structural proteins (hexon, fiber, and penton base) of the adenovirus capsid^50^. In an attempt to study the presence of anti-E3/49K and anti-pUL11 antibodies in humans we asked whether Intratect® (pooled normal human immunoglobulin from various blood donors) or Cytotect® CP (pooled human immunoglobulin from HCMV individuals) stain L23-E3/49K and L23-UL11 cells with enhanced intensity as compared to L23-wt cells. However, no differences were observed (data not shown). The findings discussed above suggest that E3/49K and pUL11 are low immunogenic. Following this line, it is not very likely that there are pre-existing antibodies in humans which interfere with E3/49K when used as modulatory transgene in clinical xenotransplantation. Low immunogenicity of E3/49K may also be of advantage to prevent induction of anti-transgene antibodies in the course after transplantation. Nevertheless, further studies should be performed to address the question whether individuals with pronounced immunological memory after HAdV or HCMV infection may not be suitable as recipients for porcine xenografts expressing E3/49K or other viral modulatory transgenes.

The availability of source pigs carrying multiple genetic modifications is a prerequisite to control human anti-pig immune responses and thus is required for further development of clinical xenotransplantation. Current strategies focus on the elimination of carbohydrate xenoantigens and expression of transgenes which control the human complement and coagulation system^4^. In an attempt to decrease human T cell responses, deletion/reduction of SLA molecules in pigs has recently been described^9,10,52^. Although this strategy resulted in reduction of T cell proliferation in vitro^9^, other immune functions like cellular cytotoxicity or B cell activation and antibody production cannot be diminished by SLA deletion. CD45 molecules are expressed by all leukocytes. Thus, it is not surprising that immunomodulation by CD45 ligands can affect various cellular functions as shown here by reduction of T, NK, and B cell reactivity. Because of this broad range of inhibitory effects, the usage of viral transgenes like E3/49K may be a promising concept to be included in genetic engineering strategies of source pigs for clinical xenotransplantation^53^.

It should be noted, however, that the in vitro experiments used for characterization of E3/49K and pUL11 represent models of the direct pathway of allo- and xenoantigen recognition. In this scenario, recipient cells are activated by direct interaction with cell surface donor MHC, co-stimulatory molecules, as well as recombinant modulatory proteins like E3/49K or pUL11. Sensitization to allo- and xenografts can also occur via the indirect pathway. In that case, graft antigens are internalized by recipient antigen presenting cells (APC), processed and presented as peptides on recipient MHC class II molecules^53^. Because of the dominant role of MHC class II molecules in the indirect pathway, it mainly triggers activation of CD4^+^ T cells. Activation via the indirect pathway is mediated solely by human–human cell interactions, making it unlikely that expression of viral proteins in source pigs will have an influence on this pathway. This is also supported by our observation that membrane expression on pig cells is required for the suppressive effects of E3/49K on T cells whereas soluble molecules were ineffective (Fig. 2 B and C). Although E3/49K may not be able to prevent sensitization of human CD4^+^ T cells via the indirect route, we assume that the cytotoxic potential of CD4^+^ effector T cells can be diminished by expression of E3/49K on cells from source pigs (Fig. 6).

## Materials and methods

### Cells

The porcine peripheral blood B cell line L23^54^ was obtained from the European Collection of Cell Cultures (London, UK). Human peripheral blood mononuclear cells (PBMC) were isolated from discarded material of normal routine apheresis samples obtained from the Department of Transfusion Medicine (Hannover Medical School). Samples were anonymized and could not be assigned to an individual donor. The local ethics committee of Hanover Medical School approved this procedure. All methods were carried out in accordance with DFG guidelines of Good Scientific Practice. Porcine PBMC (German Landrace pigs) were obtained from the Clinic for cardiac, thoracic, transplant and vascular surgery (Hannover Medical School) or from the pig herd of Friedrich Loeffler Institute (Mariensee, Germany). The mouse cell line 300–19 and the transfectant 300–19-CD45-ABC was kindly provided by M. Streuli^55^. Human and porcine PBMC were isolated using Ficoll density gradient centrifugation (Biocoll Separating Solution; Biochrom GmbH, Berlin, Germany). Human CD8^+^ T cells were isolated by depletion of HLA-DR^+^CD14^+^CD56^+^CD4^+^ cells using an antibody cocktail and MACS (Miltenyi Biotech GmbH, Bergisch Gladbach, Germany) and positively isolated after staining with CD8 by electronic cell sorting (Cell Sorting Core Facility, Hannover Medical School). Human CD4^+^ T cells were isolated by depletion of HLA-DR^+^CD14^+^CD56^+^CD8^+^ cells using an antibody cocktail and MACS. Purity of CD8^+^ T cells or CD4^+^ T cells was usually > 90%. Human CD56^+^ cells were isolated by depletion of HLA-DR^+^CD14^+^CD3^+^ or HLA-DR^+^CD14^+^CD4^+^CD8^+^ cells using an antibody cocktail and MACS. Purity of CD56^+^ cells was usually > 90%.

### Antibodies and flow cytometry

The following monoclonal antibodies (mAb) were used for cell surface staining: anti-pUL11 (clone 01)^43^, anti-E3/49K (clone 4D1)^23^, anti-pig SLA class-I (PT85A; Kingfisher Biotech/Biomol, Hamburg, Germany), anti-pig SLA class-II (MSA-3; provided by A. Saalmüller, Vienna, Austria), anti-human CD3 (OKT3; ATCC), anti-human CD4-FITC or -APC (RPA-T4; BD Biosciences; San Jose, CA, USA), anti-human CD4 (OKT4; ATCC), anti-human CD8-PE or -APC (RPA-T8; BD Biosciences), anti-human CD8 (AICD8.1, kindly provided by of B. Schraven, University of Magdeburg, Germany), anti-human CD14 (3C10; ATCC), anti-human CD56 (T199; kindly provided by T. Pietsch, University of Bonn, Germany), anti-human HLA-DR (L243; ATCC), anti-human HLA-ABC (W6/32; ATCC), anti-human CD25-PE (M-A251; BD Biosciences), anti-human CD127-PE-Cy5 (eBioRDR5; eBioscience, San Diego, California, United States), anti-human CD45RA-FITC (HI100; BD Biosciences), anti-human CD45R0-PE (UCHL1; BD Biosciences), anti-human CD19-PE (HIB19; BioLegend, San Diego, California, United States) and anti-human CD69-APC (FN50; BD Biosciences). Detection of intracellular human forkhead box-protein P3 (FoxP3) was performed using the Alexa Fluor 647 FoxP3 Flow Kit according to the manufacturer’s instructions (206D, isotype control: MOPC-21; BioLegend, San Diego, CA, USA). Purified human CD45-His protein (CD5-H52H8: ACROBiosystems, Newark, DE, USA) was used to compare the relative expression amount of E3/49K and pUL11. Bound proteins were detected using anti-human CD45-PE antibody (HI30; BD Biosciences). For purified sec49K-His the His-tagged N-terminal ectodomain of HAdV-D64 E3/49K (sec49K-His, residues 20–353) was expressed in insects cells using baculovirus as described before^33^. The UL11-Fc fusion protein (predicted extracellular domain of pUL11) was fused to the Fc domain of human IgG as described before^16^. Purified sec49K-His (0.11 µg) and UL11-Fc (1 µg) were used to study binding of E3/49K and pUL11 to CD45. Bound proteins were detected using anti-E3/49K- or anti-pUL11 mAb and the appropriate secondary antibody. Human immunoglobulin preparations pooled from various healthy blood donors (Intratect®; normal human immunoglobulin; Biotest; Dreieich, Germany) and immunoglobulins from HCMV seropositive individuals (Cytotect®; HCMV human immunoglobulin; Biotest) were used to study whether antibodies to E3/49K or pUL11 are present in human serum. Binding of unlabelled primary reagents was visualized using FITC-conjugated goat anti-mouse IgG plus IgM (Dianova; Hamburg, Germany), FITC-conjugated goat anti-rat (Dianova), FITC-conjugated goat anti-human IgG (Dianova) and FITC-conjugated goat anti-human IgM (Dianova). If not otherwise stated, Fc receptors on human cells were blocked by an incubation (prior to antibody staining) with Intratect®. Fc receptors on porcine cells were blocked using normal swine serum. Analyses were performed on a FACSCalibur flow cytometer (Becton Dickinson, San Jose, CA, USA) and data were processed by using FCS Express 7 (De Novo software, Pasadena, CA, USA).

### Generation of transfected L23 cell lines

Cloning of the HAdV-D64 E3/49K gene into the pSG5 expression vector has been described previously^56^. The plasmid construct pSG5-E3/49K and pcDNA3 plasmid without insert were used for co-transfection of L23 cells by Cell Line Nucleofector Kit (Amaxa Biosystems). A codon-optimised version of the sequence encoding UL11 from the genome of the HCMV strain Merlin^43^ using the primers 5´-ATAGAATTCTTAGAGGTCTGTCTGGGGAATCA-3´ and 5´-ATAGGATCCATGCTGTTTCGCTACATCAC-3´ and cloned into pcDNA3 (Invitrogen). The plasmid construct pcDNA3-UL11 was also used for transfection of L23 cells by Cell Line Nucleofector Kit. Transfected cells were selected with 1 mg/mL G418. For the generation of stable expressing cell lines, transfected cells expressing E3/49K or pUL11 were stained with anti-E3/49K or anti-pUL11 mAbs, isolated by fluorescence activated cell sorting (Cell Sorting Core Facility, Hannover Medical School) and cloned.

### Detection of secreted E3/49K

The amount of sec49K in the supernatant of cells was determined by binding to human PBMC followed by antibody staining and flow cytometry. L23-control and L23-E3/49K cells were also incubated for 6 h with or without the broad spectrum metalloprotease inhibitor Marimastat (10 µM; Sigma).

### Generation of cytotoxic effector cells

Cytotoxic effector cell lines were established to obtain human or porcine cytotoxic effector cells with specificity for porcine xenoantigens expressed by L23wt cells. Human purified CD4^+^ or CD8^+^ cells and porcine PBMC were cultivated in RPMI-1640 medium (Lonza, Verviers, Belgium), supplemented with 10% FCS, 2 mmol/L l-glutamine, 100 U/mL penicillin, 100 μg/mL streptomycin, 1 mmol/L sodium pyruvate, and 0.05 mmol/L β- mercaptoethanol at 5% CO_2_. Every 7 days cells were re-stimulated with irradiated L23wt cells (1:1) and 12.5 ng/ml recombinant human interleukin 2 (IL-2, Immunotools, Friesoythe, Germany). Three to four days after re-stimulation, half of the culture medium was replaced with fresh RPMI-1640 plus supplements and IL-2. Human NK cells with strong cytotoxic activity for L23wt cells were obtained by 5 to 6 days of culture of purified CD56^+^ cells in the presence of 50 ng/ml IL-2.

### Proliferation assays

Xenogeneic human anti-pig MLR experiments were performed. Thus, a total of 1 × 10^5^ human PBMC were stimulated with increasing numbers of L23 transfectants (irradiated with 30 Gray) in microtiter plates in a total volume of 200 µl culture medium (RPMI 1640 medium supplemented with 10% FCS, 50 U/mL penicillin, 4 mM L-glutamine, 50 µg/mL streptomycin, 1 mM sodium pyruvate, and 0.05 mM ß-mercaptoethanol). In some experiments human PBMC were pre-incubated with purified soluble E3/49K protein (sec49K-His, 1 µg/ml) or L23-E3/49K cell culture supernatant (sec49K). After 5 days 1 µCi/well tritiated thymidine was added and cultures were harvested after an additional 16 h incubation period. Proliferation was also studied by CFSE assays using the CellTrace™ CFSE Cell Proliferation Kit (Invitrogen; Waltham, USA). A total of 1 × 10^5^ labelled human PBMC were stimulated with 25 × 10^3^ L23 transfectants (irradiated with 30 Gray) in microtiter plates in a total volume of 200 µl culture medium. After 7 days the intensity of the CFSE staining was analysed among CD4^+^ T cells by flow cytometry. For chronic stimulation, a defined cell number of human PBMC were weekly re-stimulated by irradiated L23 transfectants (ratio 10:1) for a total of 4 weeks and used for phenotypic analysis. The cells were counted after 7, 14, 21 and 28 days and cell number was compared to the cell number at the start of that week to determine weekly increase or decrease in proliferation. Live and dead cells were discriminated by forward/side scatter characteristics. Dead cell were identified by low forward scatter intensity. Comparative studies using Propidium Iodid (BD Biosciences) and 7-AAD (BioLegend) revealed that low forward scatter is an appropriate parameter for live/dead cell discrimination in human-pig cell co-cultures. Electronic gating was performed to exclude dead cells in flow cytometry studies characterizing cell population in long-term cultures.

### Cytokine assay

The amount of cytokines induced by stimulation of 1 × 10^5^ human PBMC with 25 × 10^3^ L23 transfectants was determined in the cell culture supernatant after 24 h of co-culture by using the human Th1/Th2 Cytokine Cytometric Bead Array (BD Biosciences) according to manufacturer instructions.

### Phosphorylation assay

A total of 15–20 × 10^5^ human PBMC were stimulated for 2 h, 24 h or 48 h with 15–20 × 10^4^ L23-control, L23-UL11 or L23-E3/49K cells, respectively. The cells were then fixed using FoxP3 Fix/Perm Buffer set (BD Biosciences) for 20 min at room temperature, followed by an incubation with Perm Buffer for 15 min at room temperature. Phosphorylation at residue 505 of p56lck was detected by anti-p56lck(p505)-FITC (4/LCK-Y505), ZAP70 phosphorylation at Y319 by anti-ZAP70(p319)-Alexa Fluor488 (17A/P-ZAP70), both from BD Biosciences. Stainings of p56lck and ZAP70 were combined with CD4-APC. Stained cells were analyzed by flow cytometry.

### Assessment of cellular cytotoxicity

Briefly, a total 1 × 10^5^ porcine L23 target cells were co-cultured with human effector cells (CD4^+^ T cell lines, CD8^+^ T cell lines, IL-2 activated CD56^+^ cells) with an effector:target ratio of 1:1 in microtiter plates (Thermo Fisher Scientific, Waltham, MA, USA) and 200 µL culture medium for 2 h. Afterwards, cells were stained with the FITC Annexin V (BD Biosciences). Apoptotic L23 cells were determined by the frequency of Annexin positive cells.

### B cell stimulation and antibody production

A total of 2 × 10^5^ human PBMC were stimulated with 2 × 10^5^ L23 transfectants in microtiter plates in a total volume of 200 µl culture medium. After 24 h the expression of CD69 on CD19-positive cells was analysed. Additionally, a total of 2 × 10^5^ human PBMC were activated by 2 µg/ml CpG-B (CpG-ODN 2006; InvivoGen) and cultured with or without 2.5 µg/ml sec49K-His in 200 µl culture medium in microtiter plates. After 11 days supernatant was harvested and tested for the presence of anti-pig antibodies. Therefore, L23wt cells were incubated with supernatant and binding of antibodies was visualized using goat anti-human IgM-FITC and goat anti-human IgG-FITC.

### Statistical analysis

Statistical analysis to compare mean values obtained by two sets of data was performed by using the paired Student’s *t* test (Graphpad Prism 6 software). Comparison of three data sets was done using one-way ANOVA and Sidak’s multiple comparison test. Levels of significance are given as *P*-values (* < 0.05, ** < 0.01, *** < 0.001, **** < 0.0001).

## Data Availability

The nucleotide sequence data used for this study are available in the GenBank nucleotide sequence database under accession numbers AF271153.1 and NC_006273.2. The codon-optimised version of the UL11 nucleotice sequence from the genome of the HCMV strain Merlin^36^, which results in an unchanged amino acid sequence, is available from the corresponding author. The amino acid sequence data used for this study are available in the UniProt protein sequence database under accession numbers Q8JZH6 and Q6SWB9. The datasets generated during and/or analyzed during the current study are available from the corresponding author on reasonable request.
